# No MERS-CoV but positive influenza viruses in returning Hajj pilgrims, China, 2013–2015

**DOI:** 10.1186/s12879-017-2791-0

**Published:** 2017-11-10

**Authors:** Xuezheng Ma, Fang Liu, Lijuan Liu, Liping Zhang, Mingzhu Lu, Abuduzhayier Abudukadeer, Lingbing Wang, Feng Tian, Wei Zhen, Pengfei Yang, Kongxin Hu

**Affiliations:** 1 Institute of Health Quarantine, Chinese Academy of Inspection and Quarantine, No. 11 Ronghua South St., Beijing, 100176 China; 2 Gansu International Travel Healthcare Center, Lanzhou, No.387 Jiayuguan E. Rd, Gansu, 730001 China; 3 Xinjiang Entry-Exit Inspection and Quarantine Bureau, No. 237, Gaoxin St. Urumqi, Xinjiang, 830001 China; 4 National Institute for Disease Control and Prevention, No.155, Changbai Rd., Beijing, 102206 China; 5 Huaian Center for Disease Control and Prevention, No. 84, Zhenhuailou E Rd., Jiangsu, 223200 China

**Keywords:** Middle East Respiratory Syndrome Coronavirus (MERS-CoV), Hajj pilgrims, Respiratory viruses, China

## Abstract

**Background:**

There is global health concern that the mass movement of pilgrims to and from Mecca annually could contribute to the international spread of Middle East Respiratory Syndrome Coronavirus (MERS-CoV). In China, about 11,000 Muslim pilgrims participate in the Hajj gathering in Mecca annually. This is the first report of MERS-CoV and respiratory virus molecular screening of returning pilgrims at points of entry in China from 2013 to 2015.

**Methods and results:**

A total of 847 returning Hajj pilgrims participated in this study. The test results indicated that of the travelers, 34 tested positive for influenza A virus, 14 for influenza B virus, 4 for metapneumo virus, 2 for respiratory syncytial virus, and 3 for human coronavirus. There was a significant difference in the rates of positive and negative influenza virus tests between Hajj pilgrims with symptoms and those without. The detection rates of influenza virus were not significantly different among the three years studied, at 5.3, 6.0 and 6.3% for 2013, 2014 and 2015, respectively.

**Discussion and conclusion:**

The MERS-CoV and respiratory viruses detection results at points of entry in China from 2013 to 2015 indicated that there were no MERS-CoV infection but a 5.7% positive influenza viruses in returning Chinese pilgrims.

## Background

As of November 2015, there had been 1618 laboratory-confirmed cases of Middle East respiratory syndrome coronavirus (MERS-CoV) infection reported to the World Health Organization, and at least 579 cases had died [1, 2]. Most cases of MERS-CoV infection were reported from the Kingdom of Saudi Arabia. Annually, more than 2 million Muslim pilgrims from 184 countries attend the Hajj pilgrimage in Mecca, Saudi Arabia [3]. This mass gathering of pilgrims presents a global health risk due to the potential spread of infectious diseases, and respiratory infections are the most common infections transmitted between Hajj pilgrims [4, 5]. The viruses most commonly isolated from symptomatic patients during the Hajj pilgrimage were influenza virus and coronaviruses [4–7]. There is global concern that travelers returning from pilgrimage could contribute to the international spread of MERS-CoV. The International Health Regulations (IHR) Emergency Committee suggested that all countries perform surveillance for MERS-CoV among pilgrims during and after Hajj [8]. In China, about 11,000 Muslim pilgrims participate in the Hajj gathering in Mecca annually [9]. This is the first report of the molecular screening for MERS-CoV and respiratory viruses among returning pilgrims at points of entry in China, carried out from 2013 to 2015.

## Methods

The participants in this study were adult Hajj pilgrims who traveled in groups to Mecca, Saudi Arabia, and stayed there for 35–40 days from September to October, 2013–2015. In China, the government arranged charter flights for Hajj pilgrims to visit Mecca. Infectious disease monitoring and surveillance of foreigners travelers coming from other countries is the responsibility of AQSIQ (General Administration Quality Supervision Inspection and Quarantine of the People’s Republic of China). AQSIQ supervises 283 entry–exit ports in China, and operates on behalf of the national government. For all Chinese Hajj pilgrims, personal and flight information was recorded and a medical examination was conducted, including vaccination by a local AQSIQ office, before the trip to Mecca. Xinjiang and Gansu Province have the highest number of Hajj pilgrims visiting Mecca each year. In this study, our institute, the Chinese Academy of Inspection and Quarantine, cooperated with the Xinjiang and Gansu Entry–Exit Inspection and Quarantine Bureau. We randomly selected 847 returning pilgrims arriving at Xinjiang and Gansu airports, and asked for their consent to participate in this study. In China, every entry–exit airport has an infrared radiation thermometer, installed by AQSIQ, to screen travelers’ body temperatures. Among 847 returning pilgrims, 20 returning pilgrims triggered the alarm on passing through the infrared radiation thermometer installed at the airport to monitor travelers’ body temperature. Sixteen of these travelers were confirmed by using a clinical thermometer to have the onset of fever (>37.5 °C), and also reported a sore throat or cough on the returning flight. The remaining 831 returning pilgrims did not have a fever or other symptoms. The numbers of travelers with fever in each year were 7 (2013), 4 (2014), and 5 (2015). The mean age of all participants was 62.24 years old (SD = 6.19). In this study, 351 were females and 496 were males, and they were all Moslem. All pilgrims were asked to undergo a health examination and were vaccinated against influenza A and B in a local travel health center a week prior to departure. All participants included in this study were voluntary and signed consent forms.

For the detection of viral infection, samples included lower respiratory tract sputum, washes, and upper respiratory tract oropharyngeal swab specimens. Lower respiratory tract sputum samples were used to test for respiratory viruses during this 3-year period. All pilgrims were tested for influenza and MERS, but only those with fever were tested for the other viruses. All specimens were collected immediately when returning pilgrims arrived at each point of entry, and nucleic acid was isolated and immediately screened by real time RT-PCR for the *upE* and *ORF1a* genes of MERS-CoV provided by the World Health Organization [10, 11]. All real time PCR protocols for influenza A and B followed those used by a previous study [12]. Samples from travelers displaying a fever were also tested by real time RT-PCR for human metapneumo virus (hMPV), human respiratory syncytial virus (hRSV), and human coronaviruses HKU1, 229E, and OC43 [12]. According to the infection control and health quarantine rules at airports, the time taken between specimen collection and the reporting of results was within 4 h.

## Results

All real time RT-PCR results for MERS-CoV were negative. A total of 34 influenza A and 14 influenza B virus positive samples were detected from 2013 to 2015 (Table 1). Of these, the test results from participants with a fever indicated that 7 samples were positive for influenza A, 4 were hMPV positive, 2 were hRSV positive, and 1 participant was positive for each of HKU1, 229E, and OC43. In addition, 27 influenza A and 14 influenza B positive samples were detected from non-symptomatic travelers. No dual infections were detected. Two hypotheses were tested: (1) There is a significant difference in the positive and negative rates of influenza virus detection between Hajj pilgrims with symptoms and those without. Pearson’s Chi-square analysis indicated that there was a significant difference in the influenza virus detection rates between travelers with fever and those without symptoms (χ^2^ = 44.24, *P* = 0.00). It is of interest, although of unclear significance, that none of the influenza B positive subjects were symptomatic. (2) There is a significant difference in the rates of influenza (A and B) virus detection among the years 2013, 2014, and 2015. The rates of influenza virus detection for the years 2013, 2014, and 2015 were 5.3, 6.0, and 6.3%, respectively, and statistical analysis revealed that there was no significant difference in the rates of influenza virus detection among these three years (χ^2^ = 0.37, *P* = 0.83). All participants with fever were followed up, and none of these individuals were admitted to hospital after 15 days.

**Table 1:** The rates of respiratory virus detection among Hajj pilgrims returning to China from 2013 to 2015

Virus detection	Symptoms (χ^2^ = 44.24, *P* = 0.00)		Years (χ^2^ = 0.37, *P* = 0.83)			Total positives %
	Fever	Non-fever	2013	2014	2015	
Influenza A	7 (44%)	27 (3.2%)	20 (3.9%)	5 (6%)	9 (3.6%)	4.0% (34/847)
Influenza B	0	14 (1.7%)	7 (1.4%)	0	7 (2.8%)	1.7% (14/847)
Metapneumo virus	4 (25%)	NA	2 (0.4%)	2 (2.4%)	0	0.5% (4/847)
Respiratory syncytial virus	2 (12.5%)	NA	1 (0.2%)	1 (1.2%)	0	0.2% (2/847)
Human coronavirus HKU1	1 (6.3%)	NA	0	1 (1.2%)	0	0.1% (1/847)
Human coronavirus 229E	1 (6.3%)	NA	0	1 (1.2%)	0	0.1% (1/847)
Human coronavirus OC43	1 (6.3%)	NA	0	1 (1.2%)	0	0.1% (1/847)
Middle East respiratory syndrome coronavirus (MERS-CoV)	0	0	0	0	0	0
Total influenza (A and B) %	44% (7/16)	4.9% (41/831)	5.3% (27/511)	6.0% (5/84)	6.3% (16/252)	5.7% (48/847)
Total respiratory infection	16	41	30	11	16	6.7% (57/847)
Total	16	831	511	84	252	100% (847/847)

## Discussion

In this study, we did not detect any cases of MERS-CoV infection but respiratory virus infections including influenza A and B, hMPV, hRSV, and human coronavirus were detected among Hajj pilgrims returning to China. This result was consistent with the outcomes of similar studies of respiratory virus detection in Hajj pilgrims in France, North India, Egypt, Ghana, Saudi Arabia, and the UK [12–17]. Regarding the detection of influenza viruses, these studies reported detection rates of 7.8% in France (no vaccination) [15], 11% in North India (72% vaccination rate) [14], 14% in Egypt (20% vaccination rate) [13], 1.3% in Ghana (vaccination rate unknown) [5], and 7% in the UK (37% vaccination rate) [17]. In our study, all participants had been vaccinated against influenza virus, but 5.7% tested positive for influenza virus infection. We are unable to measure the direct impact of influenza vaccination on the resistance of Hajj pilgrims to influenza infection and further studies are required to understand the efficacy of the influenza vaccine among this population. However, increasing the rate of vaccination will help protect individuals, particularly those travelers that are most vulnerable to infection such as older adults and those that may be immunocompromised. A combination of vaccination and rapid antiviral treatment of symptomatic individuals currently offer the best strategy for the prevention and treatment of infections among Hajj pilgrims.

In this study, MERS-CoV was not detected in any of the upper respiratory swabs or sputum specimens tested. However, limiting the time taken for sample collection, the type of samples collected and the selection of participants can all affect the rates of positive detection. In previous studies, most samples were nasal swabs collected from strongly suspected symptomatic participants after they were under investigation in hospital [13–17]. However, in our study, swabs were collected from both suspected and asymptomatic returning pilgrims immediately after their arrival at airports. Our sampling design would therefore include some healthy pilgrims, thereby decreasing the rate of detection of respiratory virus infections. In addition, upper respiratory samples (nasopharyngeal swabs and sputum) have been demonstrated to have a lower MERS-CoV genome load than lower respiratory specimens such as tracheal aspirates and bronchoalveolar lavage specimens [18]. This may also have limited the detection of MERS-CoV in our study.

## Conclusions

The findings from our study demonstrate the risk of influenza infection among travelers during mass gatherings, and confirming the need for effective surveillance of imported infectious diseases at entry points into China. The Hajj pilgrimage provides a unique opportunity to test the effectiveness of different infectious disease preventive and detective measures that require a large sample size. Continued annual monitoring of MERS-CoV, influenza viruses, and other respiratory viruses (such as human rhinovirus), is needed to increase our understanding of the epidemic patterns of respiratory virus infections among Hajj pilgrims in China.
